# Dr. Maurice Howe Richardson

**Published:** 1912-09

**Authors:** 


Dr. Maurice Howe Richardson of Boston, Harvard, A. B., 1873, M. D., 1877, was found dead in bed at home, July 31, aged 60. His work in surgery was of the highest rank and he was one of the contributors to Park's and Dennis's systems.

